# Active Video Gaming and Obesity in Children 6–12 Years Old: A Systematic Review

**DOI:** 10.3390/jfmk11020192

**Published:** 2026-05-12

**Authors:** Dimitra P. Sklavou, George S. Metsios, Antonios Stavropoulos-Kalinoglou, Claire Chrysanthi Karpodini, Apostolos Vantarakis, Yiannis Koutedakis

**Affiliations:** 1 Department of Physical Education and Sport Science, University of Thessaly, 42100 Trikala, Greece; astavropoulos@uth.gr; 2 Department of Nutrition and Dietetics, University of Thessaly, 42100 Trikala, Greece; g.metsios@uth.gr; 3 Sport and Physical Activity Research Centre, Faculty of Education, Health and Wellbeing, University of Wolverhampton, Wolverhampton WV1 1LY, UK; claire_karpodini@outlook.com; 4 Medical School, University of Patras, 26504 Rio, Greece; avanta@upatras.gr

**Keywords:** childhood obesity, active video games, physical activity, BMI

## Abstract

**Objectives:** Although many governments and scientific organisations have developed strategies to combat the epidemic of childhood obesity, the unsatisfactory results thus far warrant further studies. The aim of this systematic review is to investigate the effects of active video games (AVGs) on physical activity (PA) levels and BMI (body mass index)/body composition in overweight and obese children 6–12 years of age. **Methods:** Articles were retrieved from the databases of Scopus, PubMed (MEDLINE), SPORTDiscus, and CINAHL. Thirteen articles met the inclusion criteria and were categorised according to the AVG intervention length. **Results:** AVG intervention periods of 4–12 weeks seem to moderately improve PA levels and refine BMI/body composition levels. In contrast, interventions lasting 13–24 weeks revealed encouraging results for improving PA, but had little effect on BMI/body composition levels. **Conclusions:** AVGs can generally help overweight and obese children 6–12 years of age to improve their PA levels and reduce BMI and/or improve body composition.

## 1. Introduction

Obesity is one of the largest health hazards worldwide and a major cause for morbidity and mortality [1]. The World Health Organization perceives obesity as a “global pandemic”, given that it affects approximately 30% of the world’s population [2]. High levels of childhood obesity have also been consistently found in most Westernised societies [3,4,5], including Europe [6,7], where the prevalence of overweight and obesity is 30% and 10%, respectively [8]. Greece, where the current research was conducted, is ranked third amongst the European countries in terms of overweight and obese children under 5 years old (one in eight children), and ranks second and first for children 5–9 and 10–19 years old, respectively [9].

The most concerning aspect of childhood obesity is its association with detrimental health effects. Obese children have a higher incidence of cardiovascular complications [10,11], atherosclerosis, high blood pressure [12], type 2 diabetes, hypercholesterolemia, or sleep apnoea [13] than their counterparts of normal body weight. Likewise, childhood obesity affects mental health and the prevalence of depression [14]; leads to body image dissatisfaction, low self-esteem, and eating disorders [15,16,17]; and has been linked to academic deficits [18,19].

Along with other life-style modifications [20], increasing levels of physical activity (PA) seems to be an effective formula for tackling childhood obesity and its consequences [21] both at community centres [22] and in school environments [23], although some do not offer sufficient PA opportunities [24]. However, relevant initiatives and policies have produced conflicting results, with some showing PA increases among children and concomitant weight reductions [25], whereas others revealed poor outcomes [26].

Active video games (AVGs) can be a practical and attractive form of PA for children, as they provide both fun and engaging movement-based gameplay. Given that children are familiar with new technologies, AVGs seem to be feasible in terms of adherence, as the intensity of activity ranges from low to moderate [27]. AVG players physically interact with onscreen images, usually during sport-based activities, and a camera records their body movements [28]. This active component of the videogames has the potential to increase PA levels in children who would otherwise spend time playing non-active electronic games [29,30,31]. Therefore, AVGs might offer a novel opportunity to turn sedentary behaviour into physically active screen time [32,33].

Recent systematic reviews have highlighted the positive effects of AVGs on weight control in obese/overweight children and adolescents [34,35,36,37]. Nevertheless, there are no systematic reviews focused on obese and overweight children. Considering the biological and growth differences between children and adolescents, the purpose of this systematic review was to investigate the effects of AVGs on PA levels and body mass index (BMI)/body composition in overweight and obese children 6–12 years of age. This age group is recognised as middle childhood [38]—a distinct developmental period during which children acquire the necessary cognitive, perceptual, and motor skills to understand, interact with, and effectively use active video games. The upper limit of 12 years was chosen to exclude the influence of puberty, which is accompanied by significant hormonal and somatometric changes that affect body composition and body mass index (BMI). This is the first time that AVGs have been examined in relation to this population.

## 2. Methods

**Figure 1:**
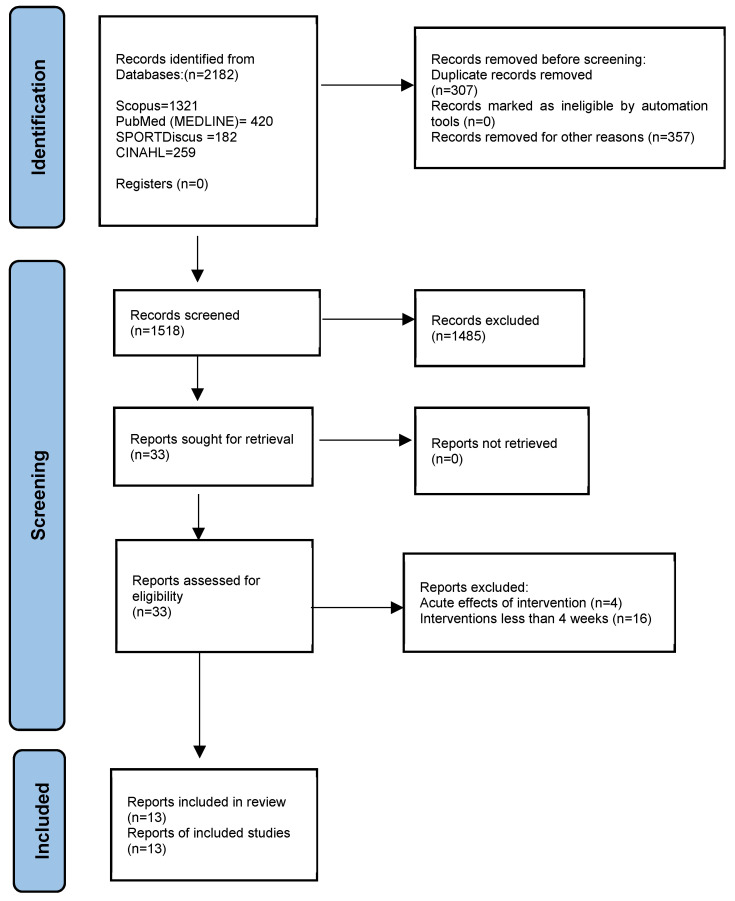
PRISMA 2020 flow diagram for new systematic reviews, which include searches of databases and registers only.

The systematic review followed the PRISMA 2020 flow diagram for new systematic reviews, which included searches of databases and registers only [39]. This review was not pre-registered in a protocol database (e.g., PROSPERO). However, it was conducted in accordance with established methodological standards, including adherence to the PRISMA guidelines, to ensure transparency and methodological rigour. The study was intended to investigate the literature without conducting a meta-analysis. The search approach and associated outcomes appear in Figure 1.

### 2.1. Search Strategy

Published articles were identified by searching electronic databases, scanning reference lists of articles, and examining references from previous relevant systematic reviews. The search strategy was applied to the Scopus, PubMed (MEDLINE), SPORTDiscus, and CINAHL databases. The search algorithms were as follows: **Population** (“child* OR “kid” OR “obes*” OR “overweight” OR “children*” OR “adolescent”) AND **Intervention** (“active video games” OR “active electronic games” OR “interactive video games’’ OR “Playstation Move” OR “exergam” OR “interactive electronic games” OR “Nintendo” OR “Wii” OR “Dance Dance Revolution” OR “fitness game” OR “physical game” OR “physical activity” OR “EyeToy” OR “egames” OR “newgeneration video games” OR “exercise” OR “AVGs*” OR “exercise video game” OR “Xbox kinect”) AND **Outcomes** (“body mass index” OR “BMI” OR “body composition” OR “body fat” OR “energy expenditure” OR “total daily energy expenditure” OR “waist circumference” OR “body fat percentage” OR “fat mass” OR “muscle mass” OR “energy metabolism” OR “metabolic equivalent” OR “bodily movement”).

Three reviewers (ASK, YK, and DPS) independently examined all studies. Titles and abstracts were screened, and relevant articles were obtained and assessed using the inclusion and exclusion criteria. The reference lists of all included studies were manually screened to identify any additional relevant articles; no further eligible studies were detected. Full text copies were subsequently obtained of all studies that met the eligibility criteria. Any disagreements about inclusion were discussed and resolved by consensus between the aforementioned reviewers.

### 2.2. Eligibility Criteria

The eligibility criteria were as follows: (1) peer-reviewed research articles in the English language; (2) articles published between January 1990 and December 2025; (3) articles that assessed children diagnosed as overweight or obese between 6 and 12 years of age; (4) articles that examined at least one type of AVG in the intervention (e.g., Nintendo Wii, Xbox 360 Kinect, Dance Revolution); (5) articles that assessed the effects of AVGs on BMI/body composition and PA in relation to overweight and/or obese children; (6) an intervention length longer than 4 weeks (even in the absence of a control group), as this is a popular lower limit for intervention protocols involving humans [40,41]; and (7) research articles of any methodological design. The exclusion criteria included non-peer-reviewed sources such as literature review articles, books, conferences proceedings, theses, book serials, and letters.

The lower limit of 1990 was chosen to capture the period during which video game technologies became more widely available, preceding the development of active video gaming (exergaming) interventions relevant to physical activity and childhood obesity [42].

Overweight and obesity in children were defined based on the body mass index (BMI)-for-age percentiles, according to the Centers for Disease Control and Prevention (CDC) growth charts. The BMI was calculated as the weight in kilograms divided by the height in metres squared (kg/m^2^) and interpreted relative to age- and sex-specific percentiles. Children were classified as overweight if their BMI was between the 85th and 94th percentile and as obese if their BMI was at or above the 95th percentile [43].

The intensity and duration of physical activity in active video game interventions were not clearly reported across the majority of included studies.

During the process of the literature search and evaluation, it was noted that the minimum and maximum intervention duration in the majority of the studies was 4 and 24 weeks, respectively. Therefore, we categorised the AVG interventions into two separate time periods—specifically, 4–12 and 13–24 weeks—in order to better assess the effects of AVG participation on PA engagement in overweight and obese children.

### 2.3. Data Extraction

Publications that met the criteria were imported into Endnote (version x7). Duplicates were removed before titles and abstracts were reviewed by ASK, YK, and DPS. Studies were excluded if (a) they did not make specific reference to AVG interventions, (b) if AVGs were employed for therapeutic purposes (cancer, diabetes, etc.), and (c) if participants were not overweight or obese or outside the age groups of interest. Only studies that reported on experimental results with mainstream AVGs and systems, such as the Wii, Sony Eye Toy, Kinect, XaviX, DDR were selected for the purposes of the current work. In addition, multicomponent interventions were excluded if the effects referring to AVGs were not separated.

### 2.4. Risk of Bias in Individual Studies

Using a 12-item TESTEX quality assessment tool (Table 1), the risk of bias of each selected study was independently rated by 2 reviewers (DPS, YK) to assess their quality [44,45,46,47,48,49,50,51,52,53,54,55,56]. For all studies, each positive item was scored one (1), while any negative item that was insufficiently reported or absent received a score of zero (0). Any disagreements in scoring were resolved by inviting a third reviewer (GSM). Lastly, the sum of all “positive” ratings for each study was calculated as the final quality score. Specifically, studies with median score of 7 and above were considered “high-quality/low risk of bias” [44,46,47,50,51,52,54], and studies that scored below 6 were considered “low-quality/high risk of bias” [45,48,49,53,55,56]. Studies with a single-group design inherently lacked randomisation and comparator groups, which was reflected in their lower TESTEX scores, and may indicate a considerable risk of bias. Nevertheless, we considered their inclusion important, as they may still provide valuable insights and contribute meaningful information to the overall understanding of the research topic.

### 2.5. Data Synthesis

Descriptive data of the encompassed studies (e.g., location of data collection, population, year) were assessed in order to define the AVGs’ characteristics. The effectiveness of the AVG interventions was examined by randomised controlled trials (RCTs) and single group trials (SGTs), which were grouped depending on (a) the duration of AVG intervention, (b) the outcomes of AVG in PA, and (c) the effects of AVG on the BMI and body composition. Both randomised controlled trials and single-group intervention studies were included to provide a more comprehensive overview of the available evidence. Given the nature of research on active video game interventions, relevant studies often employed both controlled and single-group designs, and excluding such studies would have limited the scope of the review. Differences in methodological quality between study designs were acknowledged and considered in the interpretation of the findings. The main results of the selected studies appear in Table 2.

## 3. Results

A total of 2182 articles were initially identified using our search algorithm. Out of the fourteen selected studies, eight [44,46,48,49,51,53,54,55] were conducted in the USA, one in New Zealand [45], one in Spain [50], one in Iran [52], one in Brazil [56], and one in an unknown location [47]. The sample sizes ranged from n = 4 to n = 322, with one study having a very low sample to allow for a meaningful statistical analysis to be conducted [56]. Across the 13 selected studies, a total of n = 641 participants took part in AVGs, while n = 350 acted as controls (four studies did not report a control group) [49,53,55,56]. The participants ranged from 6 to 14 years of age. Six studies adopted home AVG interventions [44,45,46,47,48,51], three used laboratory settings [52,55,56], and one was in a summer camp [49], two in recreation parks [53,54], and one in a public school [50]. Overall, n = 602 of the participants were boys, with n = 389 girls. Nine studies were RCTs [44,45,46,47,48,50,51,52,54], while the remaining four were single-group trials [49]. The duration of the interventions ranged from 5 to 24 weeks. However, in order to determine the most effective intervention length, we categorised the selected articles into those with AVG interventions lasting 4–12 weeks, a period long enough to observe meaningful physiological changes [57], and those with a duration of 13–24 weeks. Only three of the included studies [52,55,56] were conducted in a laboratory. Consequently, detailed information regarding the precise intensity and frequency of engagement with AVGs was not consistently reported. In most cases, children were encouraged to participate in AVG play at MVPA levels, as frequently as desired throughout the day.

### 3.1. AVG Interventions Lasting 4 to12 Weeks

*The effects of AVGs on PA*: Of the seven studies that included interventions lasting 4 to 12 weeks, four examined AVGs in relation to PA. Two of them reported that the AVG intervention significantly increased PA levels [47,49], and two studies revealed no effect on PA [44,55].

*The effects of AVGs on BMI/body composition:* Six out of the seven selected studies examined BMI and/or body composition. In four, BMI and body composition were found to be significantly lower following AVG interventions [47,52,53,56], whereas two studies revealed non-significant improvements [49,55].

### 3.2. AVG Interventions Lasting 13 to 24 Weeks

*The effects of AVGs on PA:* The majority of the six selected studies reported significant increases in moderate to vigorous PA [46,48,50,51], and only one study demonstrated a small but not statistically significant improvement in PA [54].

*The effects of AVGs on BMI/body composition:* Of the six studies that evaluated the effects of AVGs on BMI/body composition levels, two reported significant positive changes in BMI/body composition [48,51], one reported a small but not significant improvement [45], and the remaining three studies revealed no significant improvements [46,50,54].

## 4. Discussion

The purpose of this systematic review was to investigate the effects of AVGs on PA levels and BMI/body composition in overweight and obese children 6–12 years of age. This is the first systematic review to categorise the length of interventions, in order to better assess the effects on PA and BMI/body composition levels. The general findings suggest that AVG intervention periods of 4–12 weeks seem to be moderately adequate for improving PA levels and effective for refining BMI/body composition levels. In contrast, interventions lasting 13–24 weeks revealed encouraging results for improving PA but showed little effect on BMI/body composition levels. Therefore, AVGs may be incorporated in intervention strategies aiming at reducing childhood obesity and its detrimental effects on health and social wellbeing.

For intervention periods of 4–12 weeks, the aforementioned results may be partly explained by the fact that the move from baseline inactivity to even modest regular PA creates an early net energy deficit [58] and an associated metabolic response [59], which may be instrumental in refining BMI/body composition levels, as well as other welcome changes in metabolic physiology (e.g., improved glucose regulation). In contrast, longer AVG interventions (13–24 weeks) generally produce sustained increases in PA due to continued engagement and repeated exposure, but their impact on BMI and body composition was often limited [45], perhaps due to an insufficient cumulative energy expenditure [60]. Another reason might be the absence of behavioural change strategies, as suggested for other medical conditions [61]. Such methodologies may empower more children to participate in AVGs, allowing researchers to study their effects on daily PA and BMI levels.

In line with published systematic reviews [35,36], the present work indicates that AVG interventions have the potential to positively affect body fat in overweight or obese children. Hence, AVG interventions could be used as an effective tool for combating obesity, along with pedometers [35], smartwatches [62], etc. However, in order to achieve the expected outcome of improved PA, an intervention duration of 13–24 weeks appears to be more effective than shorter-term equivalents. This may be attributed to the fact that physical activity-related behavioural changes require relatively longer periods of time to become established and chronic [63]. In contrast, improvements in BMI seem to occur primarily during the initial 4–12 weeks of AVG engagement, perhaps due to children’s initial enthusiasm associated with their engagement in a novel form of exercise [60] and/or due to “beginners-benefits” [64]; which, nevertheless, warrant further scientific attention.

From a practical perspective, AVG interventions may be more effective when implemented regularly and at moderate-to-vigorous physical activity (MVPA) intensity, which was the most commonly reported intensity across studies. MVPA appears to be both feasible and appropriate for children with low baseline activity levels or excess body weight [65,66], as it allows for sufficient energy expenditure while maintaining engagement [67]. Additionally, a gradual progression in exercise intensity, once adherence is established, may further enhance PA outcomes and potentially BMI levels as well [68].

Furthermore, the use of unstructured free-play approaches—rather than strictly protocol-driven exercise programmes—may contribute to increased motivation and sustained engagement [35]. This may allow children to interact freely with AVGs without rigid prescriptions, and may facilitate adherence, particularly among sedentary or overweight populations.

Overall, AVG interventions may be considered as a complementary approach within broader strategies targeting childhood obesity and physical inactivity. However, given the methodological boundaries and variability across studies, further research is needed to determine the optimal characteristics and “dose” of such interventions.

### Limitations

The presented results may have certain limitations. For example, about half of the included studies used a relatively small sample of n < 50 [47,50,51,53,55,56]. A larger sample of overweight or obese children could provide more reliable information regarding the effects of AVGs in this special population. In addition, out of the 13 studies included in this research, half of them adopted home-based AVG interventions [44,45,46,47,48,51]. The data collection for the remaining studies took place in supervised environments, which may have strengthened and encouraged children’s participation.

Additionally, substantial methodological heterogeneity was observed across the included studies, such as the assessment of physical activity, BMI, and other key outcome measures, as well as in the intervention characteristics. This variability limits the comparability of findings and may affect the overall interpretation of results. Furthermore, no meta-analysis was conducted, as the primary aim was to qualitatively synthesise the available evidence.

## 5. Conclusions

Within the limitations of this review, active video games (AVGs) may contribute to improvements in physical activity (PA) levels and, to a lesser extent, body mass index (BMI) and body composition in overweight and obese children aged 6–12 years. However, the overall evidence remains heterogeneous and should be interpreted with caution. Intervention durations of approximately 4–12 weeks appear to be associated with improvements in PA levels and may have some beneficial effects on BMI and body composition, whereas longer interventions (13–24 weeks) show more consistent improvements in PA, with less clear effects on anthropometric outcomes. Further studies are needed to determine the optimal “dose” and structure of AVG-based interventions in paediatric populations.

## Figures and Tables

**Table 1:** Quality of the included studies.

	Eligibility	Random Allocation of Patients	Allocation Concealment	Groups Similar at Baseline	Blinding of Assessor	Outcomes in 85% of Patients	Intention to Treat Analysis	Statistical Comparisons	Variability	Monitoring	Intensity of Exercise	Exercise Volume and Expenditure	Total Score
Baranowski et al., 2012 [44]	1	1	0	1	0	0	0	1	1	1	1	0	7
Maddison et al., 2011 [45]	1	1	0	1	0	0	0	1	1	1	0	0	6
Lu et al., 2025 [46]	1	1	0	1	0	0	0	1	1	1	1	0	7
Murphy et al., 2009 [47]	1	1	0	1	1	1	0	1	1	1	1	1	10
Trost et al., 2014 [48]	1	1	0	1	0	0	0	1	1	1	0	0	6
Flynn et al., 2018 [49]	1	0	0	0	0	1	0	0	1	1	1	1	6
Christison et al., 2012 [53]	1	0	0	0	0	0	0	0	1	0	0	1	3
Huang et al., 2017 [55]	1	0	0	0	0	0	0	0	1	1	1	1	5
Comeras-Chueca et al., 2022 [50]	1	1	0	1	0	0	0	1	1	1	1	1	8
Staiano et al., 2018 [51]	1	1	1	1	1	1	1	1	1	1	1	1	12
Irandoust et al., 2021 [52]	1	1	0	1	0	1	0	1	1	0	0	1	7
Carrasco et al., 2013 [56]	1	0	0	0	0	1	0	0	1	0	0	1	4
Christison et al., 2016, [54]	1	1	1	1	1	0	0	1	1	0	0	0	7

**Table 2:** Main characteristics of the selected studies.

	Study and Country	Study Design	Study Description	Setting and Subjects	Exergames	Duration	Measurements	Key Findings
1	Baranowski et al., 2012, USA [44]	RCT	Examination of PA levels in children who received AVGs compared to those who received non-active videos.	Home-based *N* = 78 (38 F, 40 M); Mage = 11.3 ± 1.8 Intervention (*n* = 41); Control (*n* = 37)	Wii (Active Life-Extreme Challenge,) EA Sports Active, DDR, Wii Fit Plus, Wii Sports; inactive games: Disney Sing It-Pop Hits, Madden NFL 10, Mario Kart Wii, New Super Mario Bros, Super Mario Galaxy	12 weeks	Anthropometry, PA	No evidence that children who played AVGs were more active than those who played non-active video games.
2	Maddison et al., 2011, New Zealand [45]	RCT	Evaluation of the effectiveness of AVGs over a 6-month period on weight, body composition, PA and physical fitness in OW and OB children.	Home-based *N* = 322 (87 F, 235 M); Mage = 11.6 ± 1.1); Intervention (*n* = 160); Control (*n* = 162)	Sony Play Station, EyeToy (e.g., Play3, Kinetic, Sport, and Dance Factory)	24 weeks	Anthropometry, BMI, body fat, body composition	Intervention with AVGs has a small but clear effect on BMI and body composition in OW and OB children.
3	Lu et al., 2025, USA [46]	RCT	Examination of the effect of narrative-enhanced home-based AVGs among Black and Hispanic children with overweight and obesity.	Home-based *N* = 79 (29 F, 50 M) Age = 7–14 years Narrative and AVG group (*n* = 28); AVG Only (*n* = 25); Waitlist Control (*n* = 26)	Xbox/Kinect with six AVGs interspersed with a narrative animation Ataraxia	24 weeks	Anthropometry, PA, body composition, BMI, fasting insulin glycose, lipid panel, C-reactive protein	Participants in the narrative AVGs group demonstrated higher daily MVPA compared to the control group during the initial three months.
4	Murphy et al., 2009 [47]	RCT	Determination of the effectiveness of an AVG (DDR) in improving EDF in OW children.	Home-based *N* = 35 (17 F, 18 M) Age = 7–12 years Intervention (*n* = 23); Control (*n* = 12)	Playstation2, DDR	12 weeks	Anthropometry, BMI, aerobic fitness, FMD	Twelve weeks of DDR-use improved FMD and aerobic fitness in OW children.
5	Trost et al., 2014, USA [48]	RCT	Examination of the effects of AVGs in an evidence-based weight management programme.	Home-based *N* = 75 (41 F, 34 M) Mage = 10 ± 1.7; Intervention (*n* = 34); Control (*n* = 41)	JoinForMe treatment programme	16 weeks	Anthropometry, PA, body mass, percentage of overweight	Both groups exhibited a significant reduction in BMI, percentage overweight, and vigorous PA.
6	Flynn et al., 2018, USA [49]	Single group trial	Evaluation of whether AVG intervention would increase physical fitness, intention to exercise, self-efficacy and social influence.	Summer camp *N* = 126 (65 F, 61 M) Mage = 12.60; Intervention (*n* = 126)	Nintendo Wii and Wii Fi	6 weeks	Anthropometry, BMI, self-efficacy, PA	AVGs can enhance PA for Black and Hispanic youth who live in poverty-impacted neighbourhoods.
7	Christison et al., 2012, USA [53]	Single group trial	Evaluation of the feasibility of Exergaming for Health Programme on BMI in OW and OB children.	Recreation Park *N* = 48 (22 F, 26 M) Mage = 11.2; Intervention (*n* = 48); Pre–post study	DDR, Exerbike XG, Nintendo Wii, Makoto interactive arena, Lightspace Pay Floor, Cybex Trazer, Treadwall Xavix	10 weeks	Anthropometry, BMI	The Exergaming for Health Programme could be a useful tool for a significant drop in BMI.
8	Huang et al., 2017, USA [55]	Single group trial	Evaluation of the effects of AVGs on fitness and motivation for physical activity in OW and OB children.	Lab-based *N* = 10 (8 F, 2 M) Mage = 8 ± 1.8; Intervention (*n* = 10)	Xbox Kinetic, Nintendo Wii	8 weeks	Anthropometry, BMI, physical fitness	No significant changes were found in each component of physical fitness.
9	Comeras-Chueca et al., 2022, Spain [50]	RCT	Examination of the influence of an AVG intervention combined with multicomponent exercise on muscular fitness, PA, and motor skills in OW and OB children.	School Unit *N* = 29 (13 F, 16 M) Mage = 10.07 ± 0.87; Intervention (*n* = 21); Control (*n* = 8)	Xbox Kinetic, Nintendo Wii, BKOOL	20 weeks	Anthropometry, BMI, PA, motor skills	AVGs combined with multicomponent training seem to have positive effects on muscle fitness.
10	Staiano et al., 2018, USA [51]	RCT	Evaluation of the effectiveness of Exergaming in OW and OB children in reducing adiposity and improving cardiometabolic health.	Home-based *N* = 46 (23 F, 23 M) Mage = 11.2 ± 0.8 Intervention (*n* = 23); Control (*n* = 23)	Disneyland Adventures and Kinect Sports Season	24 weeks	Anthropometry, BMI, PA, psychosocial measures, adiposity	Exergaming at home improves children’s BMI z-score and PA levels.
11	Irandoust et al., 2021, Iran [52]	RCT	Examination of the effects of exergames and aquatic exercise on weight loss and lung function in OB children.	Lab-based *N* = 59 (59 M) **Video Game Group** (*n* = 21); Mage = 8.91 + 1.21; **Aquatic exercise Group** (*n* = 18); Mage = 9.30 + 1.30; **Control Group** (*n* = 20); Mage = 8.95 ± 1.15	Xbox Kinetic game (Wii Sports, Kinetic Ultimate Sports, Wii Fit, and just Dance)	12 weeks	Anthropometry, body composition, lung function	Use of exergaming can help OB children to lose weight, be more active, and improve lung function.
12	Carrasco et al., 2013, Brazil [56]	Single group trial	Estimation of the effect of game therapy on body composition and functional capacity of OW children who used an aerobic exercise programme on the Nintendo Wii console.	Lab-based *N* = 4 M Mage = 8.25 ± 1.5; Intervention (*n* = 4)	Wii (running, boxing karate)	5 weeks	Anthropometry, functional capacity, body mass, BMI	Significant reduction in body mass, BMI, and abdominal circumference. Functional capacity improved.
13	Christison et al., 2016, USA [54]	RCT	Examination of the effectiveness of Exergaming for Health Programme in OW and OB children.	Recreation Park *N* = 80 (46 F, 34 M) Age = 8–12 years; Intervention (*n* = 59); Control (*n* = 21)	DDR, Exerbike XG, Nintendo Wii, Makoto interactive arena, Lightspace Pay Floor, Cybex Trazer, Treadwall Xavix	24 weeks	Anthropometry, BMI, PA	Use of exergaming did not improve weight status and PA levels after a 6-month intervention programme.

Abbreviations : AVGs: active video games; DDR: Dance Dance Revolution; BMI: body mass index; PA: physical activity; EDF: endothelial dysfunction; FMD: flow-mediated dilation; OW: overweight; OB: obese; RCT: randomised controlled trial.

## Data Availability

No new data were created or analyzed in this study. Data sharing is not applicable to this article.
